# HIV, appendectomy and postoperative complications at a reference hospital in Northwest Tanzania: cross-sectional study

**DOI:** 10.1186/1742-6405-7-47

**Published:** 2010-12-29

**Authors:** Geofrey C Giiti, Humphrey D Mazigo, Jorg Heukelbach, William Mahalu

**Affiliations:** 1 Department of Surgery, Faculty of Medicine, Weill-Bugando University College of Health Sciences, P.O. Box 1464, Mwanza, Tanzania; 2 Department of Medical Parasitology and Entomology, Faculty of Medicine, Weill-Bugando University College of Health Sciences, P. O. Box 1464, Mwanza, Tanzania; 3 Department of Community Health, School of Medicine, Federal University of Ceará, Fortaleza; 4 Anton Breinl Centre for Tropical Medicine and Public Health; School of Public Health, Tropical Medicine and Rehabilitation Sciences, James Cook University, Townsville, Australia

## Abstract

**Background:**

Appendicitis is a frequent surgical emergency worldwide. The present study was conducted to determine the prevalence of HIV, and the association of infection with clinical, intraoperative and histological findings and outcome, among patients with appendicitis.

**Methods:**

We performed a cross sectional study at Weill-Bugando Medical Centre in northwest Tanzania. In total, 199 patients undergoing appendectomy were included. Demographic characteristics of patients, clinical features, laboratory, intraoperative and histopathological findings, and HIV serostatus were recorded.

**Results:**

In total, 26/199 (13.1%) were HIV-seropositive. The HIV-positive population was significantly older (mean age: 38.4 years) than the HIV-negative population (25.3 years; p < 0.001). Leukocytosis was present in 87% of seronegative patients, as compared to 34% in seropositive patients (p = 0.0001), and peritonitis was significantly more frequent among HIV-positives (31% vs. 2%; p < 0.001). The mean (SD) length of hospital stay was significantly longer in HIV-positives (7.12 ± 2.94 days vs. 4.02 ± 1.14 days; p < 0.001); 11.5% of HIV patients developed surgical site infections, as compared to 0.6% in the HIV-negative group (p = 0.004).

**Conclusion:**

HIV infections are common among patients with appendicitis in Tanzania, and are associated with severe morbidity, postoperative complications and longer hospital stays. Early diagnosis of appendicitis and prompt appendectomy are crucial in areas with high prevalence of HIV infection. Routine pre-test counseling and HIV screening for appendicitis patients is recommended to detect early cases who may benefit from HAART.

## Introduction

Appendicitis is the most frequent abdominal emergency worldwide [1-4], and also the most common cause of surgical emergency admissions in many parts of Africa [2,5]. Interestingly, the occurrence of appendicitis appears to be increasing in many low and middle income countries [6-8]. This may partly be explained by the increasing number of HIV/AIDS cases in the sub-Saharan region, as compared to high income countries [9].

In the early years of the HIV epidemic it was noted that HIV-infected patients had a higher risk of appendicitis, even beyond the risks accounted for by opportunistic infections [10]. However, little is known about the interactions between HIV infection and surgical diseases like appendicitis. Some reports have suggested that the higher occurrence of appendicitis in HIV/AIDS patients was related to the fact that the appendix is a target site for infection due to its predominant supply by terminal arteries [11]. Other studies have reported higher rates of surgical complications such as postoperative infections, impaired wound healing and higher mortality among HIV-seropositive patients [12-14]. This may lead to withholding surgery in some circumstances [15]. However, other studies did not find any difference in surgical outcomes between HIV-infected patients and the general population [16,17].

In Tanzania, limited data are available on the association between appendicitis and HIV infection, and the short-term outcome among HIV patients attending referral hospitals. In the northwest of the country, HIV prevalence in the adult population ranges from 6.7% to 10% [18]. We therefore conducted a study on patients undergoing appendectomy at a major reference hospital in northwest Tanzania.

## Materials and methods

### Study area

The study was conducted at Bugando Medical Centre (BMC) in Mwanza, north-western Tanzania. This referral hospital is situated along the southern shores of Lake Victoria and has a capacity of 900 beds. BMC is located between latitudes 2°l15'-2°45' S and longitudes 32°45'- 45° 38' E and lies at an altitude of 1140 m. The hospital serves as a referral centre for tertiary specialist care for a catchment population of approximately 13 million people from Mwanza, Mara, Kagera, Shinyanga, Tabora and Kigoma regions of Tanzania.

### Study population

We performed a cross-sectional study. All patients diagnosed with appendicitis and with indication of appendectomy presenting at BMC between August 2008 and April 2009 were eligible, irrespective of age. The inclusion criteria were the patient's willingness to give voluntary written informed consent for the study, appendectomy, and HIV testing. For patients <18 years of age, parents/guardians gave written informed consent. Patients were excluded from the study if were diagnosed to have other intraoperative findings like pelvic inflammatory disease (PID) and ectopic gestation. Patients readmitted due to late complications of appendectomy were also excluded.

### Enrolment and clinical investigation of patients

Recruitment of patients took place at casualty department. In this department, initial assessment of all patients with various infectious diseases and non-infectious disease conditions is made. The patients' information was recorded in the study questionnaires. Blood samples were taken for assessment of white blood cells; leukocytosis was defined as white blood cells count > 10,000/mm^3^, and a neutrophil shift to the left when relative neutrophil counts were >75%.

The Alvarado's scale was used to reach the diagnosis of appendicitis [19]. Patients with a score of 1-4 were considered to be very unlikely to have acute appendicitis and kept under observation. Those scored 5-6 were considered to have a diagnosis compatible with acute appendicitis, but not convincing enough to warrant appendectomy, and were regularly reviewed. Individuals with a score ≥7 were considered to have almost definite acute appendicitis, and appendectomy was indicated [19]. Patients with features of recurrent/chronic appendicitis were evaluated and recommended for operation.

### Appendectomy and postoperative follow-up

Appendectomy was carried out according to standard procedures [20]. Patients with peritonitis secondary to perforated appendicitis were subjected to laparotomy through extended midline incision [20]. During the operation, the appendix was examined macroscopically and the intraoperative findings were recorded. The resected parts of the appendix were submitted to pathology department for histopathological examination using the hematoxylin and eosin (H&E) stain [21,22].

Postoperative follow-up was made until the day of discharge from the hospital to ascertain the length of hospital stay, describe postoperative complications and mortality for both seropositive and seronegative patients. The length of hospital stay (LOS) was defined as the number of days in the wards from admission to discharge. To avoid bias, the decision to discharge patients from the ward was reached during the major ward rounds.

### HIV/AIDS testing of study participants

Patient's serostatus was screened using the Tanzania Ministry of Health and Social Welfare HIV rapid test algorithm for HIV testing. We used *SD*-*Bioline *test according to the manufacturer's instructions (Standard Diagnostics, Hagal-dong, Giheung-gu, Yongin-si, Kyonggi-do, South Korea). Briefly, 40 μL finger prick blood were applied to the sample sites on the test card. The diluents were thereafter applied as indicated by the manufacturer.

Considering the emergency characteristic of appendicitis and the possible delay due to HIV counselling, HIV testing was carried out postoperatively. Before HIV testing, the HIV/AIDS counsellor was invited to counsel consented patients. The level of immunosuppression in the HIV-seropositive patients who consented for the study was assessed by measuring the level of absolute CD4^+ ^count using FACS calibre machine (BD-Becton, Dickinson and Company, USA).

### Data management and analysis

Data were sorted out and coded before entering into a computer using Epi data 3.1 software. The stored data were then exported to SPSS for Windows version 11.5 (SPSS Inc., Chicago, IL, USA) for analysis. Association between categorical variables was tested by using Chi-squared and Fisher's exact test. The association between continuous variables was tested by using student's t-test. Odds ratios with their respective 95% confidence intervals are given.

### Ethical clearance and considerations

Ethical clearance and permission to conduct the study was obtained from the joint Bugando Medical Centre/Bugando University College of Health Sciences ethical review board (Certificate No: BREC/001/13/2008).

For patients who were coincidentally found to be HIV positive, proper post test counselling was provided and they were referred to Care and Treatment Clinic (CTC) for HIV patients at Weill-Bugando Medical Centre for further evaluation and management after being discharged from surgical wards.

## Results

A total number of 207 patients with appendicitis were admitted during the study period. Of these, five refused to participate in the study, two refused to consent for HIV test and one patient was excluded from the study because he was readmitted three days after being discharged with complication of fecal fistulae. Thus, 199 patients were included in data collection, appendectomy, HIV testing, and analysis.

### Demographic characteristics and HIV seroprevalence

In total, 110 (55.3%) were females and 89 (44.7%) males. The overall mean age (standard deviation) of patients was 27 ± 10.44 (amplitude: 7-57 years).

In total 26 (13.1%) were HIV seropositive, and 173 (87.0%) HIV-negative. The HIV-positive population was significantly older than the HIV negative population (38.4 vs. 25.3 years; p < 0.001). In the HIV-positive group, 16/26 (61.5%) were males, while in the HIV-negative group 94/173 (45.7%) were males (p = 0.491). Five (19.2%) HIV-positive patients were on Highly Active Antiretroviral Treatment (HAART). Mean CD4 counts (216 vs. 207) and mean length of hospital stays (6.4 vs. 6.0) did not differ in patients with or without HAART.

### Clinical, intraoperative and histological findings

Table 1 illustrates the clinical and intraoperative features observed in the study population with respect to HIV serostatus. Leukocytosis was a common feature in the HIV-negative group, as compared to the HIV-positives (p = 0.0001). Similarly, fever was more common among HIV-seronegative patients than in the HIV-positive population (p = 0.04).

**Table 1:** Clinical, intraoperative and histological findings of patients with appendicitis, according to HIV serostatus (n = 199).

Clinical features	HIV - serostatus		OR	95% CI	P-*value*
				
	Seropositive n (%)	Seronegative n (%)			
Migratory (Right Iliac Fossa)	19 (73.1)	153 (90.2)	0.30	0.11-0.81	0.013
Anorexia	18 (69.2)	95 (54.9)	1.85	0.76 - 4.48	0.169
Nausea/vomiting	14 (53.8)	72 (41.6)	1.64	0.72 - 3.75	0.241
Fever	12 (46.1)	115 (66.5)	0.43	0.19 - 1.00	0.044
Rebound tenderness	25 (96.2)	163 (94.2)	1.53	0.19 - 12.50	0.687
Tenderness (right lower quadrant)	23 (88.5)	160 (92.5)	0.62	0.17 - 2.35	0.482
Leukocytosis	9 (34.6)	151 (87.3)	0.08	0.03 - 0.19	0.0001
Mean leukocyte count (SD)	7.4 (1.9)	11.1 (1.4)			
Neutrophil shift	11 (42.3)	99 (57.3)	0.55	0.24 - 1.26	0.154
Mean neutrophil count (SD)	4.8 (0.96)	4.1 (1.3)			
**Intraoperative features**					
Inflamed appendix	14 (53.8)	160 (92.5)	0.10	0.04 - 0.25	0.001
Perforated appendix	8 (30.8)	6 (3.5)	2.32	0.44 - 12.16	0.307
Perforated appendix + peritonitis	2 (7.7)	4 (2.3)	18.78	5.14 - 68.55	0.001
Appendicular abscess	1 (3.8)	2 (1.2)	3.42	0.30 - 39.12	0.344
Appendicular mass	1 (3.8)	1 (0.6)	6.88	0.42 - 1.13	0.245

The mean (standard deviation) CD4^+ ^count in the HIV seropositive group was 209.31 ± 95.29 cells/μL (amplitude: 75 - 456 cells/μL). There was no association between CD4^+ ^counts (at < 200 cells/μL or at > 200 cells/μL), surgical wound infections and the length of hospital stays (p = 0.58).

Inflamed appendix was the commonest intraoperative finding in both groups. However, the frequency of peritonitis was significantly higher among HIV-positives (31%), as compared to HIV-negatives (2%; p < 0.001). Other intraoperative features are presented in Table 1. Pathohistological analysis of appendix specimens revealed that 84% of HIV seropositive patients had acute appendicitis while 66% HIV seronegative patients had acute appendicitis (*P <*0.001). In one specimen from an HIV seropositive patient, an atypical histological finding of acute appendicitis with numerous eggs of *Schistosoma *sp. in the mucosal wall was encountered.

### Outcome according to HIV serostatus

The overall mean (SD) length of hospital stay was 4.42 ± 1.83 days (range: 2-15 days). There was a highly significant association between the duration of hospital stay and HIV serostatus, with a mean length of 4.02 ± 1.14 days for HIV seronegative patients, and of 7.12 ± 2.94 days for HIV seropositive patients (p < 0.001). The longer hospital stay of HIV-positive patients could be partly explained by higher rates of complicated appendicitis observed in this group. These patients required longer follow-up before they were discharged from the hospital.

Out of the 199 individuals included, 4 (2.0%) developed surgical site infections (wound sepsis). Of these, three patients were HIV-seropositive and one patient HIV-seronegative, resulting in a frequency of 11.5% (3/26) in HIV positives and 0.6% (1/173) in HIV negatives. This indicates that surgical site infections were about 20 times more common in the case of HIV infection (P = 0.004). None of the three HIV-positive patients received HAART. All four patients recovered well and were discharged. There were no other complications noted in both groups during the time of stay in hospital. No fatal outcomes were observed during the observation period.

## Discussion

Our study shows that HIV infections were common among patients with appendicitis in a referral hospital in Tanzania. HIV patients were significantly older, and HIV infection was associated with peritonitis, postoperative complications, and longer hospital stays. Similar to other studies, leukocytosis was less frequent in HIV positive patients [16].

Previous studies suggested that the rate of acute appendicitis among HIV/AIDS patients is higher than in the general population [10,23], whereas other authors did not report any differences [16,17]. Reasons for possibly higher prevalences of appendicitis among HIV seropositive patients remain unclear, and the available literature suggests that HIV-related diseases such as lymphoma, Kaposi's sarcoma and *Mycobacterium *spp. infections may either cause or mimic appendicitis [13,23-27]. The HIV seroprevalence of 13.1% observed in our study was higher as compared to HIV prevalence of the adult population in Mwanza region, ranging from 6.7% to 10% [18]. The HIV prevalences among patients with appendicitis observed in our study were lower as compared to 16.7% from other hospital report from Cabrini Medical Centre, New York [10]. On the other hand, the HIV seroprevalence observed was slightly higher than the prevalence of 10.5% reported among hospitalized general surgical patients at another major hospital, in Eastern Tanzania [9].

Perforated appendix with peritonitis was about 15 times more frequent in the HIV seropositive group, and acute purulent appendicitis was about four times more common. These findings were consistent with previous studies [11,16,22] and call for the need of early diagnosis of appendicitis in HIV positive patients. Acute gangrenous, purulent and haemorrhagic appendicitis were the most common histological features observed among HIV-positives. The higher rates of complicated appendicitis in the HIV seropositive group may be attributed to a depressed level of cell-mediated immune response, delay in diagnosis and subsequently delay in surgical interventions.

A postoperative complication observed in the present study was surgical site infection, which was about 20 times more common in the HIV positive group. These findings were similar to other reports from other settings among HIV patients with surgical conditions [21,28-30]. The difference observed could be attributed to underlying immunosuppression in HIV seropositive patients as measured by CD4^+ ^counts.

Concerns have been raised that HIV-infected patients have longer hospital stays and greater follow-up, affecting outcomes [31,32]. In fact, in our study, HIV seropositive patients were observed to stay significantly longer in the wards as compared to HIV seronegative patients. This was similar to results of a previous study from Veteran General Hospital in Taiwan [22]. We did not observe associations between surgical site infections and the length of hospital stays. The longer mean lengths of hospital stay in HIV-positive patients with appendicitis can partly be explained by the higher rate of complicated appendicitis among HIV seropositive patients.

Among the HIV seronegative patients, one patient had an ancillary histological finding: acute appendicitis with numerous eggs of *Schistosoma mansoni *in the bowel wall. In fact, in endemic areas, *Schistosoma *species have been associated with the occurrence of various surgical conditions, including appendicitis [33]. The available evidence suggests that massive deposition of ova in the appendiceal wall may induce edema, leading to luminal obstruction and ischemia and eventually to necrosis and bacterial infection [33-35]. However, the causal relationship between schistosomiasis and the occurrence of appendicitis still remains unclear.

Our study is subject to limitations. The cross sectional nature and the small sample size of HIV positive individuals may have failed to show significant causal associations between groups. In addition, the inclusion of a single health facility which is a referral hospital may have caused selection bias, and thus interpretation of data regarding generalization should be made with care. Furthermore, the use of a single rapid antibody diagnostic test to screen patients for HIV may have resulted in false negative serostatus results in some cases.

## Conclusion

We conclude that due to vague presentation of appendicitis in HIV-positive patients and high morbidity associated with delayed diagnosis, prompt appendectomy is crucial in areas with high prevalence of HIV infection. Physicians should have a high index of suspicion of HIV/AIDS, even when leukocytosis and fever are not present. Treatment of HIV infection may decrease excess morbidity associated with infection, and thus routine pre-test counseling and HIV screening for appendicitis patients is recommended to detect early cases who may benefit from HAART.

## Competing interests

The authors declare that they have no competing interests.

## Authors' contributions

GCG and WM designed the study and participated in data collection. HDM and JH analysed the data and wrote the first draft of the manuscript. All authors contributed to the manuscript and approved its final version.
